# Analysis of exergy efficiency of a super-critical compressed carbon dioxide energy-storage system based on the orthogonal method

**DOI:** 10.1371/journal.pone.0195614

**Published:** 2018-04-10

**Authors:** Qing He, Yinping Hao, Hui Liu, Wenyi Liu

**Affiliations:** 1 School of Energy Power and Mechanical Engineering, North China Electric Power University, Beijing, China; 2 Shenhua Guohua (Beijing) Electric Power Research Institute CO.,LTD, Beijing,China; University of Akron, UNITED STATES

## Abstract

Super-critical carbon dioxide energy-storage (SC-CCES) technology is a new type of gas energy-storage technology. This paper used orthogonal method and variance analysis to make significant analysis on the factors which would affect the thermodynamics characteristics of the SC-CCES system and obtained the significant factors and interactions in the energy-storage process, the energy-release process and the whole energy-storage system. Results have shown that the interactions in the components have little influence on the energy-storage process, the energy-release process and the whole energy-storage process of the SC-CCES system, the significant factors are mainly on the characteristics of the system component itself, which will provide reference for the optimization of the thermal properties of the energy-storage system.

## Introduction

Super-critical Carbon Dioxide (SC-CO_2_) is a matter whose temperature and pressure are above the critical temperature and pressure of CO_2_. Also its physical properties are those between a liquid and gas, and it has a high diffusion coefficient, low viscosity and high density[1]. Super-critical Compressed Carbon dioxide Energy-Storage (SC-CCES) system is a novel energy-storage system that uses SC-CO_2_ to replace air as working fluid.

As a “research hotpot” in the field of energy storage, many scholars from China and overseas have carried work based on energy storage system using CO_2_ as working fluid. Ricardo Vasquez Padilla and colleagues[2] performed energy and exergy analysis of super-critical CO_2_ Brayton cycle configurations. Zhang and colleagues[3] proposed and analyzed compressed CO_2_ energy storage (CCES) systems based on Brayton cycle with hot water as the heat storage medium and did energy and exergy analysis. Morandin[4] analyzed an electrical energy-storage system in which the working fluid was CO_2_ using a heat engine cycle for energy conversion. Zhang and coworkers [5,6] analyzed a super-critical CO_2_ energy-storage system based on the Rankine cycle and Brayton cycle. Wang and coworkers [7] analyzed a liquid CO_2_ storage system based on the Brayton cycle. Liu and colleagues [8] researched the thermal characteristics of SC-CO_2_ and trans-critical CO_2_ energy-storage systems.

The studies detailed above analyzed the mechanism of the conversion and loss of energy-storage systems from different viewpoints. These include how the thermodynamic characteristics of the energy-storage system were influenced by parameters in the process of energy storage and release, and how the interactions among these parameters influenced on the thermodynamic characteristics. In any study of thermal characteristics of SC-CCES systems, comprehensive thermal analysis would result in a huge workload because of the many design parameters involved (such as tedious repetitive computations and calculations) and the interactions between them.

The orthogonal analysis method[9] uses an orthogonal table to arrange and analyze multi-factorial experiments. Based on orthogonal characteristics, some experimental points which have a uniform distribution and good characteristics are selected to do the experiment. A normalized orthogonal table can be used to balance a collocation of multiple factors and obtain an optimum combination of factor levels rapidly and accurately. In this way, fewer experiments and simulations are needed to analyze the significance of each factor on the optimization goal[10,11].

We undertook numerical simulation analysis on the exergy efficiency of a SC-CCES system using the orthogonal method. We carried out variance analysis on the results of the orthogonal design, quantified design parameters, and calculated the influence exergy efficiency to determine significant and non-significant factors, which will provide reference for the optimization of energy-storage system.

## System description

### Working principle of the SC-CCES system

**Fig 1:**
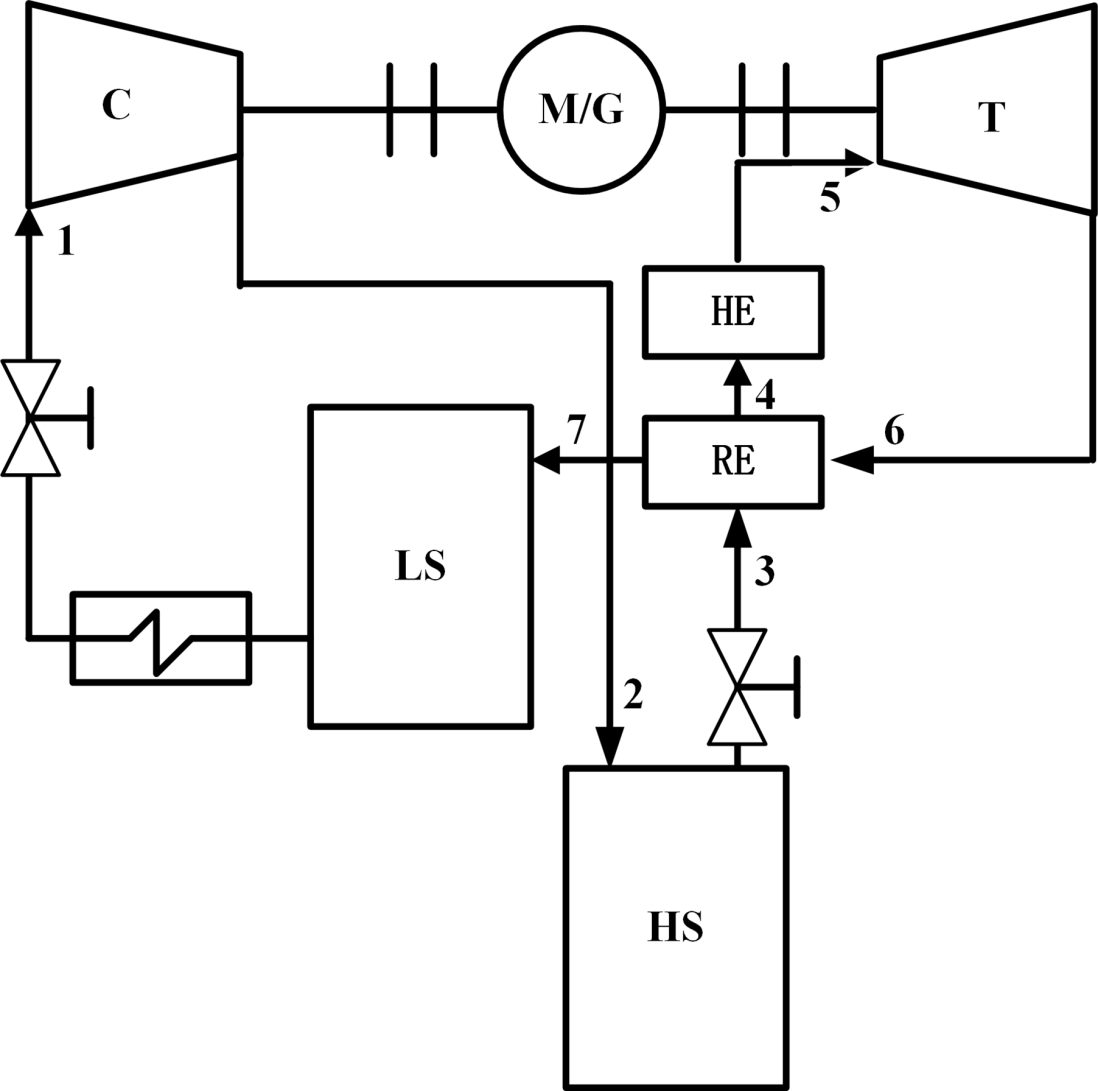
Schematic of the SC-CCES system. C = compressor, M = motor, G = generator, T = expansion turbine, HE = heater, RE = regenerator, LS = low pressure reservoir, HS = high pressure reservoir.

The SC-CCES system used two saline aquifers as storage reservoirs was a closed energy storage cycle[8]. The first reservoir was a low-pressure reservoir used to store CO_2_ exhausted from the turbine. The second reservoir was a high-pressure reservoir used to store CO_2_ from the compressor. A schematic of the system is shown in Fig 1. The thermodynamic design parameters of the system in the working process are shown in S1 Table.

The working principle was based on the energy-storage process and the energy-release process. In the energy-storage process, the working fluid (SC-CO_2_) was stored in a low-pressure reservoir cooled to ambient temperature by a cooler. It was depressurized through a throttle valve, and then fed into a compressor to complete the boosting process (marked “1–2” in Fig 1). The high pressure CO_2_ (marked “2” in Fig 1) was stored in a high-pressure gas-storage reservoir.

In the energy-release process, the high-pressure CO_2_ (marked “2” in Fig 1) was regulated to a certain pressure through a valve. Then, it was transported to a recuperator system to absorb the heat exhausted from the turbine (marked “3–4”in Fig 1), heated by the heater (marked “4–5” in Fig 1) and then fed into a turbine to work (marked “5–6” in Fig 1), the exhausted CO_2_ (marked “6” in Fig 1) entered the regenerator to heat CO_2_ from the pressure of the high reservoir (marked “3–4” in Fig 1). CO_2_ (marked “7” in Fig 1) released as waste heat was stored in the low-pressure reservoir.

### Analysis of exergy efficiency

#### Compressor model

The isentropic efficiency of compressor *η*_*c*_ [12,13] is

$$\eta_{c}=\frac{h_{1s}{}^{"}-h_{1}{}^{'}}{h_{1}{}^{"}-h_{1}{}^{'}},$$ (1)

where *h*_1_' is the inlet enthalpy(kJ/kg); *h*_1*s*_" is the outlet enthalpy during isentropic compression (kJ/kg); and *h*_1_" is the real enthalpy during compression (kJ/kg).

During isentropic compression, the entropies of the initial and final states are identical, i.e.

$$S_{1s}{}^{"}=S_{1}{}^{'},$$ (2)

where *S*_1_'is inlet entropy((kJ/(kg·K)), and *S*_1*s*_" is outlet entropies during isentropic compression (kJ/(kg·K)).

According to the state equation

$$h_{1s}{}^{"}=f(S_{1s}{}^{"},p_{1}{}^{"}).$$ (3)

Using Eqs (1) and (3), the outlet enthalpy *h*_1_" can be obtained. Hence, the power consumed in the compressor *W*_*c*_ is

$$W_{c}=h_{1}{}^{"}-h_{1}{}^{'}.$$ (4)

#### Turbine model

The calculation method for the actual expansion is the same as that for compression. The isentropic efficiency of turbine *η*_*T*_ [12,13] is

$$\eta_{T}=\frac{h_{2}{}^{'}-h_{2s}{}^{"}}{h_{2}{}^{'}-h_{2}{}^{"}},$$ (5)

where *h*_2_' is the inlet enthalpy (kJ/kg); *h*_2*s*_" is the outlet enthalpy during isentropic expansion (kJ/kg); and *h*_2_" is the real enthalpy during expansion (kJ/kg).

During isentropic expansion, the entropies of the initial and final states are identical, i.e.

$$S_{2s}{}^{"}=S_{2}{}^{'},$$ (6)

where *S*_2_' is the inlet entropies (kJ/(kg·K)); and *S*_2*s*_" is the outlet entropies during isentropic compression (kJ/(kg·K)).

According to the state equation

$$h_{2s}{}^{"}=f(S_{2s}{}^{"},p_{2}{}^{"}),$$ (7)

Using Eqs (5) and (7), the outlet enthalpy *h*_2_" can be obtained. Hence, the power consumed in the turbine *W*_*T*_ is

$$W_{T}=h_{2}{}^{'}-h_{2}{}^{"},$$ (8)

#### Storage model

CO_2_ was injected into an underground gas-storage chamber, which was a saline reservoir. The CO_2_ pressure had to be at least as high as the initial groundwater pressure in the reservoir. The groundwater pressure *P*_*hs*_ can be determined by

$$P_{hs}=\rho_{w}gh,$$ (9)

where *ρ*_*w*_ is the density of the reservoir groundwater (kg/m^3^); *g* is the acceleration of gravity (N/kg); and *h* is the depth of reservoir (m).

Due to the influence of the geothermal gradient, the temperature of the reservoir will increase with depth. Using values for the surface temperature and geothermal gradient, the underground temperature as a function of depth can be determined by

$$T=T_{s}+Gh,$$ (10)

where *T*_*s*_ is the surface temperature (K); and *G* is the geothermal gradient (K/km).

### Heat exchanger model

When CO_2_ is under the super-critical state, properties such as density, specific heat and viscosity undergo drastic variations with temperature variation, which will have considerable effects on system performance. Therefore, the heat-exchanging process must be divided into adequately small sections, so that the variations in properties in each section are so small that we can assume them to be constant[14].

In the inner cooler of the compressor, CO_2_ was on the hot-stream side and water was on the cold-stream side. We assumed that the temperature drop Δ*T* on the hot-stream side was known, which was divided into *N* equal difference. Hence, the heat transfer for each step *i* and mass flow rate of water were calculated from the following equations

$${\overset{•}{Q}}_{\mathrm{he},i}={\dot{m}}_{co_{2}}\cdot C_{{P,\mathrm{co}}_{2},i}^{}\cdot \left(T_{co_{2},i+1}-T_{co_{2},i}\right),$$ (11)

$${\overset{•}{Q}}_{\mathrm{he},i}={\dot{m}}_{w}\cdot C_{P,w,i}^{}\cdot \left(T_{w,i+1}-T_{w,i}\right),$$ (12)

$${\dot{m}}_{w}=\frac{\sum_{i}^{N}{\dot{Q}}_{\mathrm{he},i}}{\left(h_{w,\mathrm{out}}-h_{w,\mathrm{in}}\right)},$$ (13)

where ${\dot{Q}}_{\mathrm{he},i}$ is the *i* segment heat (kJ/s); ${\dot{m}}_{{\mathrm{CO}}_{2}}$ is the mass flow rate of CO_2_ (kg/s); $C_{{P,\mathrm{CO}}_{2},i}^{}$ is the *i* segment constant-pressure specific heat of CO_2_ (kJ/(kg·K)); $T_{{\mathrm{CO}}_{2},i+1}$ is the *i* segment outlet temperature of CO_2_ (K); $T_{{\mathrm{CO}}_{2},i}$ is the *i* segment CO_2_ inlet temperature CO_2_ (K); *T*_w, *i*+1_ is the *i* segment outlet temperature of the cooling water (K); *T*_w, *i*_ is the *i* segment inlet temperature of the cooling water (K); ${\dot{m}}_{w}$ is mass flow of cooling water (kg/s); *C*_p, w, *i*_ is the *i* segment constant pressure specific heat of the cooling water (kJ/(kg·K)); *N* is the divided section number; *h*_*w*,*out*_ is the outlet enthalpy of the cooling water (kJ/kg); and *h*_*w*,*in*_ is the inlet enthalpy of the cooling water (kJ/kg).

In the regenerator, the working fluid was CO_2_ on the hot-stream side and cold-stream side, and the flow on both sides was identical. According to the characteristics of the regenerator, the enthalpy change for the hot-stream was divided into *N* equal differences, and we could obtain the *i* segment outlet temperature on the cold stream side of the regenerator using

$$T_{{\mathrm{CO}}_{2},i+1}=T_{{\mathrm{CO}}_{2},i}+\frac{\Delta h_{\mathrm{re},i}}{{\dot{m}}_{{\mathrm{CO}}_{2}}\cdot C_{{P,\mathrm{CO}}_{2},i}^{}},$$ (14)

where Δ*h*_re,*I*_ is the *i* segment heat enthalpy (kJ/kg).

### Heater model

We assumed that heat from the heater of the SC-CCES system was all the heat released from the combustion of natural gas. Ignoring the heat loss in the heater, the main energy loss in the heater was heat loss of the combustion of natural gas. Hence, the heater efficiency was given by

$$\eta_{h}=\frac{\overset{•}{Q_{h}}}{m_{ng}\cdot H_{L}},$$ (15)

where *η*_h_ is the heater efficiency; *m*_*ng*_ is gas consumption per minute(m^3^/kg); $\overset{•}{Q_{h}}$ is heat absorption of CO_2_ per unit time(kJ/kg); and *H*_*L*_ is low calorific value of gas(kJ/m^3^).

Exergy efficiency *η*_ex_ [15] is based on the second law of thermodynamics, which can evaluate the degree of energy conversion from the viewpoints of quality and quantity of the equipment, process, or the system employed in the energy conversion. Hence, we adopted *η*_*ex*_ as the evaluation criterion in the SC-CCES system.

In the energy-storage process, exergy efficiency *η*_ex1_ is

$$\eta_{\mathrm{ex}1}=\frac{E_{3}}{W_{c}},$$ (16)

where *E*_3_ is the outlet exergy values of high-pressure gas-storage chamber after the valve (kW); and *W*_c_ is the power consumed in the compressor (kW).

In the energy-release process, exergy efficiency *η*_ex2_ is

$$\eta_{\mathrm{ex}2}=\frac{w_{T}}{E_{Q}+E_{3}},$$ (17)

where *W*_T_ is the power consumed in the turbine (kW); *E*_*Q*_ is the inlet exergy value of the heater (kW).

In the whole energy-storage process, exergy efficiency *η*_ex_ is

$$\eta_{\mathrm{ex}}=\frac{W_{T}}{E_{Q}+W_{C}},$$ (18)

### Orthogonal design for the SC-CCES system

The SC-CCES system comprised two parts: the energy-storage process and the energy-release process. The key technologies were those for the compressor and expansion turbine, heater, and gas-storage chamber. The main parameters which have the greatest effect on the thermodynamics of the system are: adiabatic efficiency of the compressor; outlet pressure of the compressor, inlet pressure of the expansion turbine, inlet temperature of the expansion turbine, adiabatic efficiency of the expansion turbine; differences in the regenerator used.

In the design and actual operation of the SC-CCES system, the charge and discharge capacity of the system can be controlled by adjusting the CO_2_ injected into the high-pressure gas–storage chamber and the low-pressure gas-storage chamber, and the change of the operation pressure; the change of the regenerator difference; and the change of combustion efficiency can also influence the operation efficiency of the compressor and the expansion turbine. Therefore, conducting a parametric analysis to understand the effects of these various parameters on the performance of the system is essential and parameters range of variance are shown in S2 Table [16,17].

We selected the key technology parameters from the design parameters of the SC-CCES system for investigation: adiabatic efficiency of the compressor (A); inlet pressure of the compressor (B); pressure of high-pressure reservoir (C); regenerator difference (D); adiabatic efficiency of the expansion turbine (E); combustion efficiency (F). To investigate the influence of these factors on exergy efficiency, the lever of each factor was assumed according to that shown in S3 Table.

In the energy-storage process, we took three three-level factors (adiabatic efficiency of the compressor (A), inlet pressure of the compressor (B), pressure of the high-pressure reservoir (C)) and three interactions (adiabatic efficiency of the compressor and inlet pressure of the compressor (A×B), adiabatic efficiency of the compressor and pressure of the high-pressure reservoir (A×C), inlet pressure of the compressor and pressure of high-pressure reservoir (B×C)), using the orthogonal design shown in S4 Table.

In the energy-release process, we took four three-level factors (pressure of the high -pressure reservoir (C), regenerator difference (D), adiabatic efficiency of the expansion turbine (E) and combustion efficiency (F)) and three interactions (adiabatic efficiency of the expansion turbine and combustion efficiency (E×F), adiabatic efficiency of the expansion turbine and pressure of high-pressure reservoir (E×C), adiabatic efficiency of the expansion turbine and regenerator difference (E×D)), using the orthogonal design shown in S5 Table.

In the whole energy-storage process of the SC-CCES system, we took six three-level factors and three interactions (adiabatic efficiency of the compressor and inlet pressure of the compressor (A×B), adiabatic efficiency of the compressor and adiabatic efficiency of the expansion turbine (A×E), inlet pressure of the compressor and regenerator difference (B×D) using the orthogonal design shown in S6 Table.

## Results and discussion

According to the design schemes of S4–S6 Tables, we took numerical calculations on the energy-storage process, the energy-release process and the whole energy-storage process and then obtained exergy efficiency of each process. According to the arrangement of the orthogonal design experiment shown in S7 Table, we analyzed the exergy efficiency and obtained the results. The results are shown in S8 Table.

From the statistical analysis shown in S8 Table, we conclude that the exergy efficiency in the energy-storage process is much higher than that in the energy-release process. This is because the definition for exergy efficiency is different for energy-storage and energy-release. Exergy efficiency in the energy-storage process is defined as the ratio of the exergy value output by the pressure of high-pressure reservoir and exergy value input by the compressor. Exergy efficiency in the energy-release process is defined as the ratio of the work output by the expansion turbine and heat exergy value and the pressure exergy value input by the energy-released system.

We undertook variance analysis according to the orthogonal design and obtained calculation results. The specific calculation formula of the orthogonal design and variance analysis[18] is

$${\bar{K}}_{ij}=\frac{r}{N}K_{ij},$$ (19)

$$\bar{y}=\frac{1}{N}\sum_{i=1}^{n}y_{i},$$ (20)

$$S_{j}=\frac{N}{r}{\sum_{i=1}^{r}\left({\bar{K}}_{ij}-\bar{y}\right)}^{2},$$ (21)

$$\upsilon_{j}=r-1,$$ (22)

$${\bar{S}}_{j}=\frac{S_{j}}{\upsilon_{j}},$$ (23)

where *r* is the level number of the factors; *N* is the number of simulations; *K*_*ij*_ is the simulation results of the factor *j* and the *i*th level; $\bar{y}$ is the average calculation results; ${\bar{K}}_{ij}$ is the bias squares for *Sj*; and ${\bar{S}}_{j}$ is the mean sum of squares.

In the variance analysis of orthogonal design results, due to the existence of an error column, if ${\bar{S}}_{j}<{\bar{S}}_{e}$, it should convert *S*_*j*_ into *S*_*e*_, which is the square sum of error sum e^Δ^ and becomes $S_{e^{\Delta }}$, so the calculation formula of *F* is

$$F=\frac{s_{j}/v_{j}}{s_{e^{\Delta }}/v_{e^{\Delta }}},$$ (24)

where *S* is the bias squares of the factors; *υ*_*j*_ is the freedom degree of the factors, when ${\bar{S}}_{j}<{\bar{S}}_{e}$, $S_{e^{\Delta }}$ is the sum of *S*_*j*_ and *S*_*e*_, $\upsilon_{e^{\Delta }}$ is the sum of *υ*_*j*_ and *υ*_*e*_.

It will make a compare between the calculation *F* and *F*_*α*_ referred to the value at significance level *α*, if *F*> *F*_*α*_, it means factors significantly. We can use this method to judge the significance produced by factors and the effects of interactions on simulation results. The results are shown in S9–S11 Tables.

Assuming that, at a significance level *α* = 0.05, using the F-distribution table, we can obtain: F_0.05_(28) = 4.46,F_0.05_(4,8) = 3.84,F_0.05_(2,10) = 4.10,F_0.05_(4,10) = 3.48, F_0.05_(2,16) = 3.63, F_0.05_(4,16) = 3.01.

Using S9 Table and comparison with the F value are shown that, in the energy-storage process of the SC-CCES system, the two significant factors that influenced exergy efficiency are adiabatic efficiency of the compressor and inlet pressure of the compressor. We also find that the following interactions are non-significant: compressor efficiency an inlet pressure of the compressor; efficiency of the compressor and pressure of the high-pressure reservoir; and inlet pressure of the compressor and pressure of the high-pressure reservoir.

S10 Table shows that in the energy-release process, pressure of the high-pressure reservoir, regenerator difference, adiabatic efficiency of the expansion turbine, and the combustion efficiency are the significant factors on exergy efficiency, and that the interaction between these factors is non-significant. Also, of the significant factors affected exergy efficiency in the energy-release process, the most significant is the pressure of high-pressure reservoir and the second is adiabatic efficiency of the expansion turbine, and the third is combustion efficiency; the least significant factor is regenerator difference.

S11 Table shows that, in the whole energy-storage process, adiabatic efficiency of the compressor, inlet pressure of the compressor, pressure of the high- pressure reservoir, regenerator difference, adiabatic efficiency of the expansion turbine and combustion efficiency are on the exergy efficiency, and that the interaction between these factors is non-significant. Furthermore, of the significant factors affecting the exergy efficiency of the whole energy-storage process, the influence of the adiabatic efficiency of the expansion turbine, combustion efficiency and inlet pressure of the compressor is much greater than that of the other factors.

In the energy-release process of the system, throttle valve is used to reduce pressure to ensure the constant inlet pressure of the expansion turbine, and works at the rated working condition, which results in a higher exergy loss and reduces the system efficiency in the SC-CCES system[19,20]. It can be seen from S10 and S11 Tables that pressure of the high-pressure reservoir has a significant influence on the exergy efficiency of the energy-release process and the whole energy-storage process of the SC-CCE system.

## Conclusions

To analyze the effect of design parameters and the interactions between them on the thermodynamic properties in a SC-CCES system, we assessed the thermodynamic properties using the orthogonal method. We also undertook variance analysis on the results of the orthogonal method,

We find that, in the energy-storage process of the SC-CCES system, the following interactions are non-significant: compressor efficiency and inlet pressure of the compressor; efficiency and pressure of the high-pressure reservoir. Also the interaction between the inlet pressure of the compressor and pressure of the high-pressure reservoir are non-significant factors.

In the energy-release process of the SC-CCES system, pressure of the high-pressure reservoir, regenerator difference, expansion turbine adiabatic efficiency, and that the interaction between these factors are non-significant.

In the whole energy-storage process of the SC-CCES system, interactions between the factors mentioned above are all non-significant. We also find that the significant factors which influence the thermodynamic characteristics of the system are not the interactions of the components but the characteristics of the system itself, the optimization direction of the SC-CCES system should focus on the characteristics optimization of components.

## Supporting information

S1 Fig Schematic of the SC-CCES system. (DOCX)

S1 Table Thermodynamic parameters of the SC-CCES system. (DOCX)

S2 Table The parameters of the SC-CCES system. (DOCX)

S3 Table Factor level in the orthogonal design. (DOCX)

S4 Table Orthogonal design of the energy-storage process. (DOCX)

S5 Table Orthogonal design of the energy-release process. (DOCX)

S6 Table Orthogonal design of the whole energy-storage process. (DOCX)

S7 Table Orthogonal design of experimental scheme. (DOCX)

S8 Table Exergy efficiency analysis using the orthogonal method. (DOCX)

S9 Table Variance analysis of exergy efficiency of the energy-storage process. (DOCX)

S10 Table Variance analysis of exergy efficiency of the energy-release process. (DOCX)

S11 Table Variance analysis of exergy efficiency of the whole energy-storage process. (DOCX)
