# Supplementary material for: Analysis of exergy efficiency of a super-critical compressed carbon dioxide energy-storage system based on the orthogonal method
Source: PLoS One. 2018 Apr 10;13(4):e0195614. doi: 10.1371/journal.pone.0195614 (PMC5892920; doi:10.1371/journal.pone.0195614)
Supplement: S1 Fig — (DOCX) [file pone.0195614.s001.docx]

Fig1. Schematic of the SC-CCES system

C=compressor, M=motor, G=generator, T= expansion turbine, HE=heater, RE=regenerator, LS=low pressure reservoir, HS=high pressure reservoir
