# Supplementary material for: Analysis of exergy efficiency of a super-critical compressed carbon dioxide energy-storage system based on the orthogonal method
Source: PLoS One. 2018 Apr 10;13(4):e0195614. doi: 10.1371/journal.pone.0195614 (PMC5892920; doi:10.1371/journal.pone.0195614)
Supplement: S1 Table — (DOCX) [file pone.0195614.s002.docx]

Table1 Thermodynamic parameters of the SC-CCES system

| Stream | Material stream | | | *T* (K) | *P* (MPa) |
| --- | --- | --- | --- | --- | --- |
| 1 | | CO_2_ | 308 | | 7.40 |
| 2 | | CO_2_ | 433 | | 40.00 |
| 3 | | CO_2_ | 382 | | 20.00 |
| 4 | | CO_2_ | 699 | | 20.00 |
| 5 | | CO_2_ | 873 | | 20.00 |
| 6 | | CO_2_ | 764 | | 8.00 |
| 7 | | CO_2_ | 387 | | 8.00 |
