# Supplementary material for: Analysis of exergy efficiency of a super-critical compressed carbon dioxide energy-storage system based on the orthogonal method
Source: PLoS One. 2018 Apr 10;13(4):e0195614. doi: 10.1371/journal.pone.0195614 (PMC5892920; doi:10.1371/journal.pone.0195614)
Supplement: S2 Table — (DOCX) [file pone.0195614.s003.docx]

Table 2 the parameters of the SC-CCES system

| Parameters | Range of variation | Units |
| --- | --- | --- |
| Pressure of the high-pressure reservoir | 40-56 | MPa |
| Inlet pressure of the compressor | 2-10 | MPa |
| Regenerator difference | 3-9 | K |
| Combustion efficiency | 0.85-0.95 | - |
| Adiabatic efficiency of the compressor | 0.80-0.92 | - |
| Adiabatic efficiency of the expansion turbine | 0.80-0.92 | - |
