# Supplementary material for: Analysis of exergy efficiency of a super-critical compressed carbon dioxide energy-storage system based on the orthogonal method
Source: PLoS One. 2018 Apr 10;13(4):e0195614. doi: 10.1371/journal.pone.0195614 (PMC5892920; doi:10.1371/journal.pone.0195614)
Supplement: S3 Table — (DOCX) [file pone.0195614.s004.docx]

Table 3 Factor level in the orthogonal design

|  | | Factor | | | | | | | | | | |  |
| --- | --- | --- | --- | --- | --- | --- | --- | --- | --- | --- | --- | --- | --- |
| Lev-er | Adiabatic efficiency of the compressor A (%) | | Inlet pressure  of the  compressor  B (MPa) | Pressure of high-pressure reservoir  C (MPa) | | Regenerator difference  D (K) | | Adiabatic efficiency of the expansion turbine E (%) | | | Combuston efficiency F (%) |  |  |
| 1 | | 80 | 8 | | 40 | | 3 | | 80 | 85 | | |  |
| 2 | | 85 | 9 | | 45 | | 6 | | 85 | 90 | | |  |
| 3 | | | 92 | 10 | | 50 | | 9 | | 92 | 95 | | |
