# Supplementary material for: Analysis of exergy efficiency of a super-critical compressed carbon dioxide energy-storage system based on the orthogonal method
Source: PLoS One. 2018 Apr 10;13(4):e0195614. doi: 10.1371/journal.pone.0195614 (PMC5892920; doi:10.1371/journal.pone.0195614)
Supplement: S5 Table — (DOCX) [file pone.0195614.s006.docx]

Table 5 Orthogonal design of the energy-release process

| 1 | 2 | 3 | 4 | 5 | 6 | 7 | 8 | 9 | 10 | 11 | 12 | 13 |
| --- | --- | --- | --- | --- | --- | --- | --- | --- | --- | --- | --- | --- |
| E | F | E×F | E×F | C | E×C | E×C | E×D | E×D | D | error | error | error |
