# Supplementary material for: Analysis of exergy efficiency of a super-critical compressed carbon dioxide energy-storage system based on the orthogonal method
Source: PLoS One. 2018 Apr 10;13(4):e0195614. doi: 10.1371/journal.pone.0195614 (PMC5892920; doi:10.1371/journal.pone.0195614)
Supplement: S6 Table — (DOCX) [file pone.0195614.s007.docx]

Table 6 Orthogonal design of the whole energy-storage process

| 1 | 2 | 3 | 4 | 5 | 6 | 7 | 8 | 9 | 10 | 11 | 12 | 13 |
| --- | --- | --- | --- | --- | --- | --- | --- | --- | --- | --- | --- | --- |
| B | A | A×B | A×B | D | B×D  A×E | B×D | error | E | C | error | A×E | F |
