# Supplementary material for: Analysis of exergy efficiency of a super-critical compressed carbon dioxide energy-storage system based on the orthogonal method
Source: PLoS One. 2018 Apr 10;13(4):e0195614. doi: 10.1371/journal.pone.0195614 (PMC5892920; doi:10.1371/journal.pone.0195614)
Supplement: S7 Table — (DOCX) [file pone.0195614.s008.docx]

Table 7 Orthogonal design of experimental scheme

| Test number | Factor | | | | | | | | | | | | |
| --- | --- | --- | --- | --- | --- | --- | --- | --- | --- | --- | --- | --- | --- |
|  | 1 | 2 | 3 | 4 | 5 | 6 | 7 | 8 | 9 | 10 | 11 | 12 | 13 |
| 1 | 1 | 1 | 1 | 1 | 1 | 1 | 1 | 1 | 1 | 1 | 1 | 1 | 1 |
| 2 | 1 | 1 | 1 | 1 | 2 | 2 | 2 | 2 | 2 | 2 | 2 | 2 | 2 |
| 3 | 1 | 1 | 1 | 1 | 3 | 3 | 3 | 3 | 3 | 3 | 3 | 3 | 3 |
| 4 | 1 | 2 | 2 | 2 | 1 | 1 | 1 | 2 | 2 | 2 | 3 | 3 | 3 |
| 5 | 1 | 2 | 2 | 2 | 2 | 2 | 2 | 3 | 3 | 3 | 1 | 1 | 1 |
| 6 | 1 | 2 | 2 | 2 | 3 | 3 | 3 | 1 | 1 | 1 | 2 | 2 | 2 |
| 7 | 1 | 3 | 3 | 3 | 1 | 1 | 1 | 3 | 3 | 3 | 2 | 2 | 2 |
| 8 | 1 | 3 | 3 | 3 | 2 | 2 | 2 | 1 | 1 | 1 | 3 | 3 | 3 |
| 9 | 1 | 3 | 3 | 3 | 3 | 3 | 3 | 2 | 2 | 2 | 1 | 1 | 1 |
| 10 | 2 | 1 | 1 | 3 | 1 | 2 | 3 | 1 | 2 | 3 | 1 | 2 | 3 |
| 11 | 2 | 1 | 2 | 3 | 2 | 3 | 1 | 2 | 3 | 1 | 2 | 3 | 1 |
| 12 | 2 | 1 | 3 | 3 | 3 | 1 | 2 | 3 | 1 | 2 | 3 | 1 | 2 |
| 13 | 2 | 2 | 1 | 1 | 1 | 2 | 3 | 2 | 3 | 1 | 3 | 1 | 2 |
| 14 | 2 | 2 | 2 | 1 | 2 | 3 | 1 | 3 | 1 | 2 | 1 | 2 | 3 |
| 15 | 2 | 2 | 3 | 1 | 3 | 1 | 2 | 1 | 2 | 3 | 2 | 3 | 1 |
| 16 | 2 | 3 | 1 | 2 | 1 | 2 | 3 | 3 | 1 | 2 | 2 | 3 | 1 |
| 17 | 2 | 3 | 2 | 2 | 2 | 3 | 1 | 1 | 2 | 3 | 3 | 1 | 2 |
| 18 | 2 | 3 | 3 | 2 | 3 | 1 | 2 | 2 | 3 | 1 | 1 | 2 | 3 |
| 19 | 3 | 1 | 3 | 2 | 1 | 3 | 2 | 1 | 3 | 2 | 1 | 3 | 2 |
| 20 | 3 | 1 | 3 | 2 | 2 | 1 | 3 | 2 | 1 | 3 | 2 | 1 | 3 |
| 21 | 3 | 1 | 3 | 2 | 3 | 2 | 1 | 3 | 2 | 1 | 3 | 2 | 1 |
| 22 | 3 | 2 | 1 | 3 | 1 | 3 | 2 | 2 | 1 | 3 | 3 | 2 | 1 |
| 23 | 3 | 2 | 1 | 3 | 2 | 1 | 3 | 3 | 2 | 1 | 1 | 3 | 2 |
| 24 | 3 | 2 | 1 | 3 | 3 | 2 | 1 | 1 | 3 | 2 | 2 | 1 | 3 |
| 25 | 3 | 3 | 2 | 1 | 1 | 3 | 2 | 3 | 2 | 1 | 2 | 1 | 3 |
| 26 | 3 | 3 | 2 | 1 | 2 | 1 | 3 | 1 | 3 | 2 | 3 | 2 | 1 |
| 27 | 3 | 3 | 2 | 1 | 3 | 2 | 1 | 2 | 1 | 3 | 1 | 3 | 2 |
