# Supplementary material for: Analysis of exergy efficiency of a super-critical compressed carbon dioxide energy-storage system based on the orthogonal method
Source: PLoS One. 2018 Apr 10;13(4):e0195614. doi: 10.1371/journal.pone.0195614 (PMC5892920; doi:10.1371/journal.pone.0195614)
Supplement: S8 Table — (DOCX) [file pone.0195614.s009.docx]

Table 8 Exergy efficiency analysis using the orthogonal method

| Number | Exergy efficiency in the energy -storage process (%) | | | Exergy efficiency in the energy -release process (%) | | Exergy efficiency in the whole energy storage -process (%) | Number | | Exergy efficiency in the energy -storage process (%) | | Exergy efficiency in the energy -release process (%) | | Exergy efficiency in the whole energy- storage -process (%) | |
| --- | --- | --- | --- | --- | --- | --- | --- | --- | --- | --- | --- | --- | --- | --- |
| 1 | | 81.66 | | | 26.90 | 47.75 | 15 | 89.08 | | 34.59 | | 55.66 | |  |
| 2 | | | 81.22 | | 30.06 | 53.02 | 16 | 91.71 | | 29.25 | | 54.83 | |  |
| 3 | | | 80.68 | | 32.22 | 58.46 | 17 | 91.78 | | 32.80 | | 59.90 | |  |
| 4 | | | 88.21 | | 27.30 | 54.68 | 18 | 91.64 | | 35.80 | | 60.06 | |  |
| 5 | | | 88.04 | | 30.60 | 55.83 | 19 | 84.78 | | 29.81 | | 59.44 | |  |
| 6 | | | 87.69 | | 33.42 | 49.45 | 20 | 84.59 | | 33.22 | | 57.88 | |  |
| 7 | | | 90.63 | | 27.67 | 60.02 | 21 | 84.26 | | 36.14 | | 49.97 | |  |
| 8 | | | 90.59 | | 31.65 | 53.30 | 22 | 90.86 | | 30.30 | | 52.00 | |  |
| 9 | | | 90.34 | | 34.00 | 52.96 | 23 | 90.94 | | 34.47 | | 52.73 | |  |
| 10 | | | 83.04 | | 28.47 | 60.31 | 24 | 90.83 | | 36.89 | | 58.95 | |  |
| 11 | | | 82.71 | | 31.69 | 54.01 | 25 | 93.07 | | 31.35 | | 58.27 | |  |
| 12 | | | 89.08 | | 33.91 | 53.47 | 26 | 93.28 | | 35.06 | | 58.45 | |  |
| 13 | | | 89.39 | | 28.85 | 58.05 | 27 | 93.27 | | 37.59 | | 57.33 | |  |
| 14 | | | 89.32 | | 32.26 | 57.21 | - | - | | - | | - | |  |
