# Supplementary material for: Analysis of exergy efficiency of a super-critical compressed carbon dioxide energy-storage system based on the orthogonal method
Source: PLoS One. 2018 Apr 10;13(4):e0195614. doi: 10.1371/journal.pone.0195614 (PMC5892920; doi:10.1371/journal.pone.0195614)
Supplement: S9 Table — (DOCX) [file pone.0195614.s010.docx]

Table 9 Variance analysis of exergy efficiency of the energy-storage process

| Sources of variance | Bias squares,  *S_j_* | Freedom degree, *f_j_* | Sum of mean  square,*$\overline{S}_{j}$* | *F* |
| --- | --- | --- | --- | --- |
| A | 4.20×10^1^ | 2 | 2.10×10^1^ | 4.94×10^1^ |
| B | 3.24×10^2^ | 2 | 1.62×10^2^ | 3.81×10^2^ |
| C | 1.20×10^0^ | 2 | 6.00×10^-1^ | 1.41×10^0^ |
| A×B | 3.20×10^0^ | 4 | 8.00×10^-1^ | 1.88×10^0^ |
| A×C | 2.89×10^0^ | 4 | 7.23×10^-1^ | 1.70×10^0^ |
| B×C | 4.67×10^0^ | 4 | 1.17×10^0^ | 2.75×10^0^ |
| error | 3.40×10^0^ | 8 | 4.25×10^-1^ | - |
| *e*^Δ^ | 3.40×10^0^ | 8 | 4.25×10^-1^ | - |
