# Supplementary material for: Analysis of exergy efficiency of a super-critical compressed carbon dioxide energy-storage system based on the orthogonal method
Source: PLoS One. 2018 Apr 10;13(4):e0195614. doi: 10.1371/journal.pone.0195614 (PMC5892920; doi:10.1371/journal.pone.0195614)
Supplement: S10 Table — (DOCX) [file pone.0195614.s011.docx]

Table 10 Variance analysis of exergy efficiency of the energy-release process

| Sources of variance | Bias squares,  *S_j_* | Freedom degree, *f_j_* | Sum of mean  square,*$\overline{S}_{j}$* | *F* |
| --- | --- | --- | --- | --- |
| C | 1.68×10^2^ | 2 | 8.38×10^1^ | 2.09×10^4^ |
| D | 4.50×10^-1^ | 2 | 2.3×10^-1^ | 5.75×10^1^ |
| E | 5.36×10^1^ | 2 | 2.68×10^-1^ | 6.71×10^3^ |
| F | 9.03×10^0^ | 2 | 4.52×10^0^ | 1.13×10^3^ |
| E×F | 1.00×10^-2^ | 4 | 3.00×10^-3^ | - |
| E×D | 9.00×10^-2^ | 4 | 2.00×10^-2^ | 5.00×10^0^ |
| E×C | 5.00×10^-2^ | 4 | 1.00×10^-2^ | 2.50×10^0^ |
| error | 3.00×10^-2^ | 6 | 5.00×10^-3^ | - |
| *e*^Δ^ | 4.00×10^-2^ | 10 | 4.00×10^-3^ | - |
