# Supplementary material for: Analysis of exergy efficiency of a super-critical compressed carbon dioxide energy-storage system based on the orthogonal method
Source: PLoS One. 2018 Apr 10;13(4):e0195614. doi: 10.1371/journal.pone.0195614 (PMC5892920; doi:10.1371/journal.pone.0195614)
Supplement: S11 Table — (DOCX) [file pone.0195614.s012.docx]

Table 11 Variance analysis of exergy efficiency of the whole energy-storage process

| Sources of variance | Bias squares,  *S_j_* | Freedom degree, *f_j_* | Sum of mean  square,*$\overline{S}_{j}$* | *F* |
| --- | --- | --- | --- | --- |
| A | 3.17×10^1^ | 2 | 1.59×10^1^ | 1.06×10^2^ |
| B | 4.59×10^1^ | 2 | 2.30×10^1^ | 1.53×10^2^ |
| C | 6.39×10^1^ | 2 | 3.20×10^1^ | 2.13×10^2^ |
| D | 4.71×10^0^ | 2 | 2.36×10^0^ | 1.57×10^2^ |
| E | 9.16×10^1^ | 2 | 4.58×10^1^ | 3.05×10^2^ |
| F | 7.95×10^1^ | 2 | 3.98×10^1^ | 2.65×10^2^ |
| A×B | 1.20×10^-1^ | 4 | 3.00×10^-2^ | - |
| B×D | 5.10×10^-1^ | 4 | 1.3×10^-1^ | - |
| A×E | 1.00×10^-1^ | 4 | 3.00×10^-2^ | - |
| error | 1.70×10^1^ | 4 | 4.30×10^-1^ | - |
| *e*^Δ^ | 2.43×10^1^ | 16 | 1.5×10^-1^ | - |
